# Supplementary material for: A deep state-space analysis framework for cancer patient latent state estimation and classification from EHR time-series data
Source: PLoS One. 2026 Jan 30;21(1):e0341003. doi: 10.1371/journal.pone.0341003 (PMC12858016; doi:10.1371/journal.pone.0341003)
Supplement: S1 Appendix — This appendix provides an overview of the analysis dataset, including its composition and key characteristics. (DOCX) [file pone.0341003.s001.docx]

# **Overview of the Analysis Dataset**

The distribution of the number of time steps (i.e., sequence lengths) in the patient records targeted in this study is shown in S1 Fig. 1. S1 Table 1 lists the 50 lab test items included in the dataset described in Section II: Dataset of this study. For each item, the frequency of occurrences of abnormally low, abnormally high, and normal values across all patients and all time points is shown in S1 Fig. 2 and 3. S1 Fig. 2 presents 25 of the 50 items, while S1 Fig. 3 shows the remaining items. Abnormally low values ​​and abnormally high values ​​are in accordance with the standards of Kyoto University Hospital.


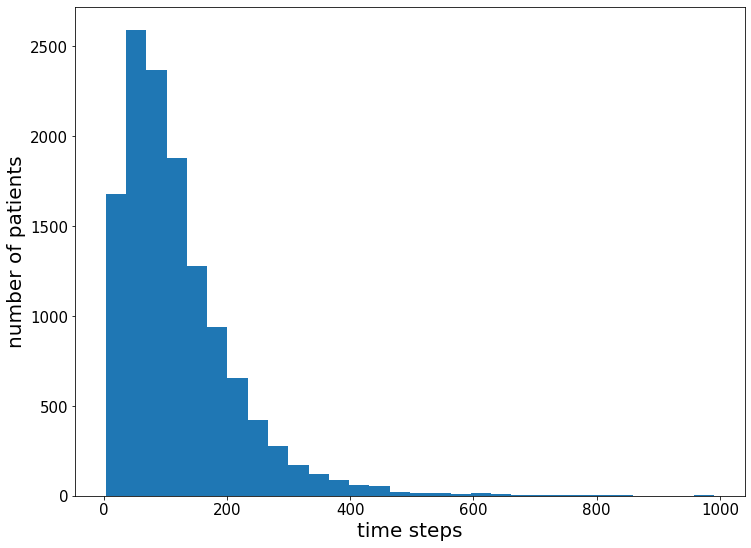


S1 Fig. 1: Number of time steps in records per patient. The vertical axis of this figure is the number of patients, and the horizontal axis is the number of time steps.

S1 Table 1. List of laboratory test items in the dataset

| Albumin | Creatinine | Sodium (Na) | Monocyte | Fibrinogen |
| --- | --- | --- | --- | --- |
| CRP (C-Reactive Protein) | White Blood Cell Count (WBC) | Mean Corpuscular Hemoglobin (MCH) | D-Dimer (FDP・D-Dimer) | LAP (Leucine Aminopeptidase) |
| Mean Corpuscular Hemoglobin Concentration (MCHC) | GOT (AST, Aspartate Aminotransferase) | Mean Corpuscular Volume (MCV) | Triglyceride (TG) | Glucose (Plasma) |
| Red Blood Cell Count (RBC) | Total Protein (TP) | Reticulocyte Count [‰] | Eosinophil | CEA (Carcinoembryonic Antigen) |
| Hematocrit | Cholesterol (TC) | Chloride (Cl) | Neutrophil | Inorganic Phosphorus (IP) |
| Glucose (Serum) | GPT (ALT, Alanine Aminotransferase) | Cholinesterase (CHE) | Prothrombin Time Activity (PT Activity) | Magnesium (Mg) |
| γ-GTP (Gamma-Glutamyl Transferase) | Platelet Count | Uric Acid (UA) | Total Bilirubin (TB) | Direct Bilirubin (DB) |
| Hemoglobin | Calcium (Ca) | Amylase | Activated Partial Thromboplastin Time (APTT) | Hemoglobin A1c (JDS) |
| Creatine Kinase (CK) | Blood Urea Nitrogen (BUN) | Lymphocyte | INR (International Normalized Ratio) | Stab Form Neutrophil |
| LDH (Lactate Dehydrogenase) | Alkaline Phosphatase (ALP) | Potassium (K) | Segmented Neutrophil | CYFRA (Cytokeratin 19 Fragment) |


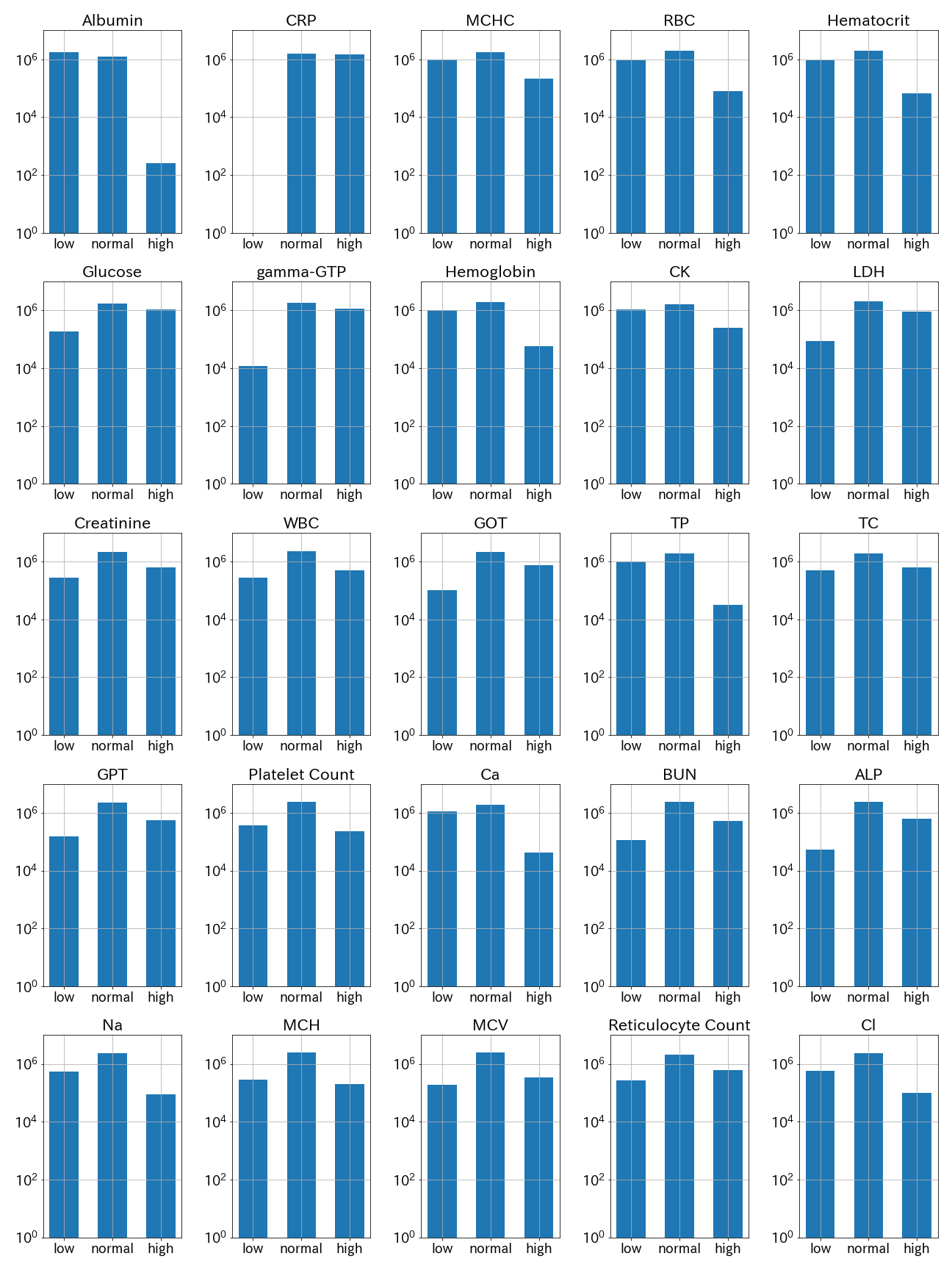


S1 Fig. 2: Frequencies of occurrences of abnormally low, high, and normal values in the dataset. The histograms of the first 25 in 50 items are shown.


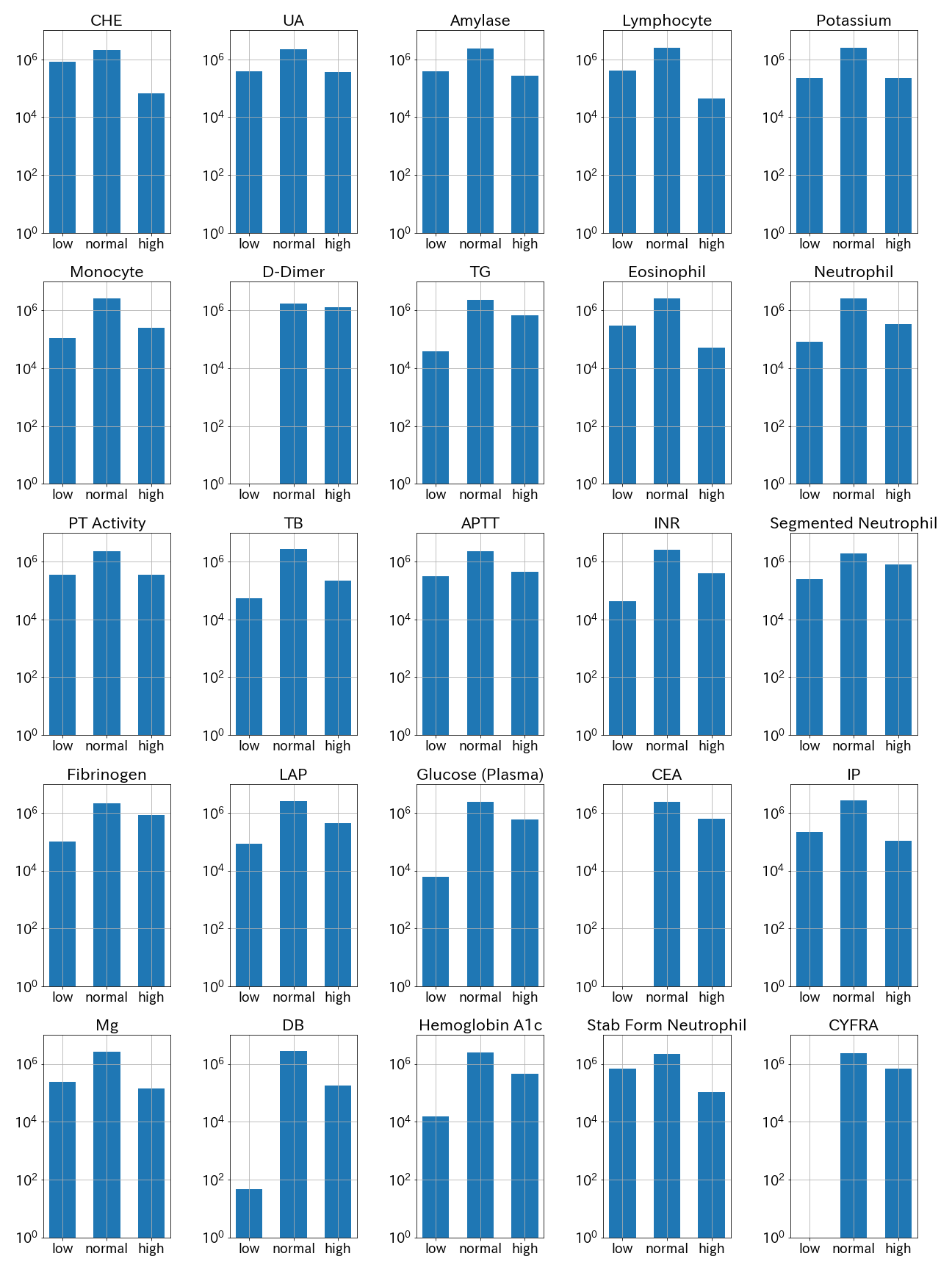


S1 Fig. 3: Frequencies of occurrences of abnormally low, high, and normal values in the dataset. The histograms of the rest 25 in 50 items are shown.

| **Drug Administered** | **Number of Patients** | **Deaths** | **Survivors** |
| --- | --- | --- | --- |
| Nivolumab | 404 | 205 | 199 |
| Trastuzumab | 267 | 73 | 194 |
| Cisplatin | 3799 | 1775 | 2024 |
| Bicalutamide | 1064 | 148 | 916 |
| Imatinib | 125 | 9 | 116 |
| Osimertinib | 103 | 21 | 82 |
| Afatinib | 99 | 40 | 59 |
| Erlotinib | 385 | 264 | 121 |

S1: Table. 2 : Patient Outcomes by Administered Drug
