# Supplementary material for: A deep state-space analysis framework for cancer patient latent state estimation and classification from EHR time-series data
Source: PLoS One. 2026 Jan 30;21(1):e0341003. doi: 10.1371/journal.pone.0341003 (PMC12858016; doi:10.1371/journal.pone.0341003)
Supplement: S2 Appendix — This appendix details the model architecture and the hyperparameters used in the study. (DOCX) [file pone.0341003.s002.docx]

# **Model Architecture and Hyperparameters**

The neural network $p_{\theta}\left( x_{t}|z_{t} \right)$ from state space $z_{t}$ to observation $x_{t}$ consists of two fully connected layers, and the neural network $p_{\theta}\left( z_{t}|z_{t-1} \right)$ from state space (time t) to state space (time t+1) used two fully connected layers. In addition, the neural network $q_{\phi}\left( z_{t}|x \right)$from the observation to the state space uses two layers: a fully connected layer and a long short-term memory (LSTM) layer.

Since the estimation results vary depending on the hyperparameters, we tried the grid search with the parameters in Table 4, qualitatively captured the mortality and survival states, and analyzed the results obtained with lr=0.005 and dim=8, which are the easiest to interpret.

Table 4. Parameter settings for deep state-space model

| Parameter name | Meaning | Parameter tried |
| --- | --- | --- |
| Dim | Number of dimensions of latent state | 2, 4, 8, 16 |
| Lr | Learning rate | 0.005, 0.01 |

**Configuration of Comparative Methods**

To demonstrate that the performance differences are derived from the model's capability to capture non-linear temporal dynamics rather than implementation discrepancies, we standardized the latent dimension to $d=8$ for all methods before applying UMAP visualization. The specific implementations were as follows:

1. Principal Component Analysis (PCA)

PCA was employed as a linear baseline. We extracted the top 8 principal components from the preprocessed EHR data to form the latent state representation.

1. Variational Autoencoder (VAE)

The VAE was implemented to capture non-linear relationships without temporal dynamics. The architecture and training settings were:

- **Architecture:** The encoder consisted of a 3-layer Multilayer Perceptron (MLP) with a 30-dimensional hidden layer and ReLU activation functions, mapping the input to a 8-dimensional latent space. The decoder mirrored this structure, consisting of a 3-layer MLP with a 30-dimensional hidden layer and ReLU activation functions, reconstructing the input from the latent space.
- **Prior Distribution:** A standard normal distribution $N(0, I)$ was used as the prior.
- **Loss Function:** The model was trained by minimizing the sum of the reconstruction loss and the Kullback-Leibler (KL) divergence, with the KL term weight set to $\alpha = 1$.
- **Training:** The model was trained using the Adam optimizer with a batch size of 256 for 50 epochs.

1. **Linear State-Space Model (LSSM)**

The LSSM was implemented to evaluate the impact of non-linearity in time-series modeling.

- **Architecture:** The structure of the LSSM was identical to that of the proposed Deep State-Space Model (DSSM), with the exception that all neural networks (MLPs) within the transition and observation models were replaced with linear transformation matrices.
- **Settings:** All other hyperparameters, including the dimension of the latent state ($d=8$), loss function calculation, and optimization procedures, were kept consistent with those of the DSSM to strictly isolate the effect of non-linear modeling capabilities.
