# Supplementary material for: A deep state-space analysis framework for cancer patient latent state estimation and classification from EHR time-series data
Source: PLoS One. 2026 Jan 30;21(1):e0341003. doi: 10.1371/journal.pone.0341003 (PMC12858016; doi:10.1371/journal.pone.0341003)
Supplement: S1 Table — This table shows the probability of transitioning between each pair of clusters identified in this study. (DOCX) [file pone.0341003.s003.docx]

S1 Table 1: Percentage of transitions between clusters for all patients (number of transitions)

|  |  | After transition | | |
| --- | --- | --- | --- | --- |
|  |  | I | II | III |
| Before transition | I | 26.27%  (432,946 persons) | 1.31%  (21,751 persons) | 0.03%  (552 persons) |
|  | II | 1.54%  (25,506 persons) | 41.00%  (675,787 persons) | 3.17%  (52,397 persons) |
|  | III | 0.00%  (139 persons) | 3.37%  (55,646 persons) | 23.25%  (383,315 persons) |

S1 Table 2: Percentage of transitions between clusters of dead patients (number of transitions)

|  |  | After transition | | |
| --- | --- | --- | --- | --- |
|  |  | I | II | III |
| Before transition | I | 36.45%  (245,627 persons) | 1.43%  (9,642 persons) | 0.02%  (199 persons) |
|  | II | 1.85%  (12,478 persons) | 38.21%  (257,510 persons) | 2.49%  (16,797 persons) |
|  | III | 0.00%  (45 persons) | 2.80%  (18,880 persons) | 16.71%  (112,624 persons) |

S1 Table 3: Percentage of transitions between clusters of surviving patients (number of transitions)

|  |  | After transition | | |
| --- | --- | --- | --- | --- |
|  |  | I | II | III |
| Before transition | I | 19.22%  (187,319 persons) | 1.24%  (12,109 persons) | 0.03%  (353 persons) |
|  | II | 1.33%  (13,028 persons) | 42.93%  (418,277 persons) | 3.65%  (35,600 persons) |
|  | III | 0.00%  (94 persons) | 3.77%  (36,766 persons) | 27.78%  (270,691 persons) |
