# Supplementary figures and images for: A deep state-space analysis framework for cancer patient latent state estimation and classification from EHR time-series data
Source: PLoS One. 2026 Jan 30;21(1):e0341003. doi: 10.1371/journal.pone.0341003 (PMC12858016; doi:10.1371/journal.pone.0341003)

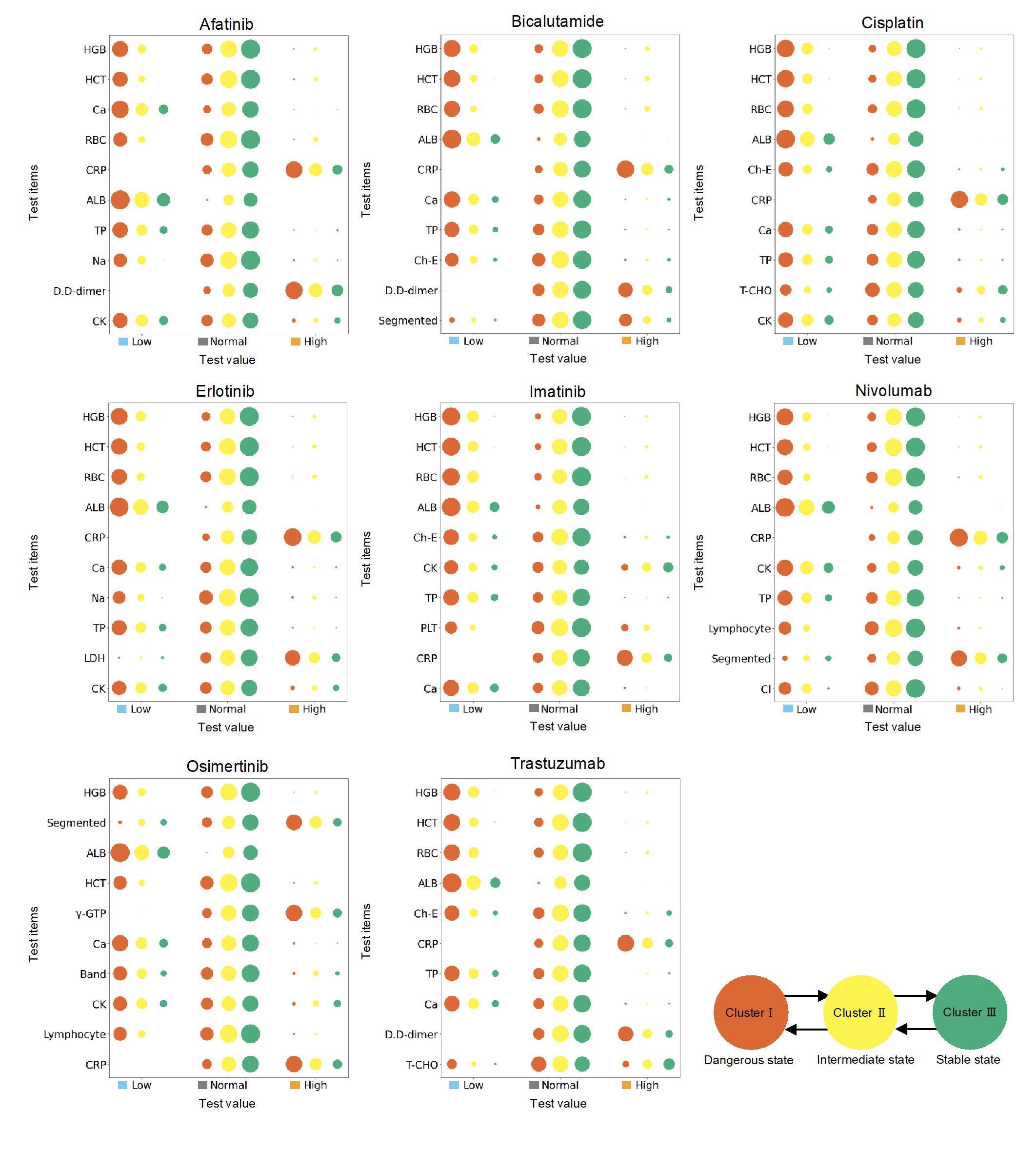

Supplement: S1 Fig — This figure shows the percentage of test items classified as abnormal in each cluster, highlighting differences in abnormal result rates among clusters. (TIF) [file pone.0341003.s004.tif]
