# Supplementary material for: A deep state-space analysis framework for cancer patient latent state estimation and classification from EHR time-series data
Source: PLoS One. 2026 Jan 30;21(1):e0341003. doi: 10.1371/journal.pone.0341003 (PMC12858016; doi:10.1371/journal.pone.0341003)
Supplement: S3 Appendix — This appendix validates the robustness of a Deep State-Space Model against missing data in NCAR and MNAR scenarios using Langevin dynamics-based synthetic simulations. (PDF) [file pone.0341003.s005.pdf]

S5 Appendix. Robustness of DeepSSM to Missing Data Rates

Yuji Okamoto, Aya Nakamura, Ryosuke Kojima, Eiichiro Uchino, Yohei Mineharu, Yohei Harada, Mayumi Kamada, Minoru Sakuragi, Manabu Muto, Motoko Yanagita, Yasushi Okuno

Article Title: A Deep state-space Analysis Framework for Cancer Patient Latent State Estimation and Classification from EHR Time-Series Data

1 Introduction

The proposed framework, which combines a Deep State-Space Model (DSSM) with k-means clustering, is designed to capture latent patient state transitions from time-series clinical laboratory data. However, real-world Electronic Health Records (EHR) exhibit extremely high missing rates. Therefore, validating the robustness of the proposed method in such data-sparse environments is essential.

Since ground truth (complete data) does not exist for real-world data, it is difficult to rigorously evaluate the impact of missing values on model training and estimation using real data alone.

In this study, to quantitatively evaluate the robustness of our method, we generated synthetic time-series data using simulations based on Stochastic Differential Equations (SDEs). By artificially introducing missing values equivalent to those found in real data into this complete dataset, we verified the impact of missingness on the accuracy of latent state estimation.

2 Methods

2.1 Creation of Comparative Datasets

In this section, we describe the SDE model constructed to mimic the transition dynamics between discrete Markov states in a continuous space, and the details of the synthetic dataset ("Toy Data") generated using this model. The synthetic data used here is designed to be as close as possible to the setting of this study, consisting of a combination of time-series data that can be globally interpreted as two different discrete Markov processes.

2.1.1 Langevin Dynamics Based on Potential Fields

To represent the behavior of a Markov chain described by a transition probability matrix A as the motion of particles in a continuous 2D space, we introduce the Langevin equation based on a potential function $U(\mathbf{x})$. The time evolution of the particle position $\mathbf{x}_t \in \mathbb{R}^2$ is assumed to follow the Itô stochastic differential equation:

$$d\mathbf{x}_t = -\nabla U(\mathbf{x}_t)dt + \sqrt{2\beta^{-1}}d\mathbf{W}_t \tag{1}$$

Here, $-\nabla U(\mathbf{x}_t)$ is the drift term derived from the potential gradient, and $\mathbf{W}_t$ represents a standard Wiener process. $\beta^{-1} = k_B T$ is the temperature parameter, which determines the noise intensity in the system.

Correspondence between Potential Function Construction and Markov Matrix A The potential function $U(\mathbf{x})$ is designed to physically implement the dynamic characteristics of the Markov chain transition probability matrix A as a potential landscape in continuous space. In this model, $U(\mathbf{x})$ is defined by taking the negative logarithm of a superposition of Gaussian basis functions.

Specifically, considering a set of states $\mathcal{S} = \{1, \ldots, M\}$ and a set of state pairs with significant transition probabilities $\mathcal{E} = \{(i,j) \mid i < j, \Phi_{ij} > 0\}$, the potential function is constructed as follows:

$$U(\mathbf{x}) = -\log\left(\sum_{i\in\mathcal{S}} \pi_i G(\mathbf{x}; \mathbf{c}_i) + \sum_{(i,j)\in\mathcal{E}} w_{ij}^{\text{bridge}} G(\mathbf{x}; \mathbf{c}_{ij})\right) \tag{2}$$

Here, $G(\mathbf{x}; \mathbf{c}) = \exp\left(-\frac{\|\mathbf{x}-\mathbf{c}\|^2}{2\sigma^2}\right)$ is a Gaussian basis function (unnormalized) with center $\mathbf{c}$ and variance σ^2. Each term has the following physical meaning:

The first term, representing the basins of attraction, governs the stability of each Markov state i. These basins are centered at fixed coordinates $\mathbf{c}_i$ on the 2D plane corresponding to each discrete state. The depth of the potential well for each state is determined by the weight π_i, which corresponds to the stationary distribution vector π of the transition probability matrix A (satisfying $A^T\pi = \pi$ and $\sum \pi_i = 1$). Consequently, states with higher stationary probabilities π_i form deeper potential wells, thereby increasing the residence time of particles within those states and effectively modeling their stability.

The second term introduces bridge components that act as saddle points to regulate the transition barriers between communicating states i and j. These components are centered at the midpoint of the transition path, defined as $\mathbf{c}_{ij} = (\mathbf{c}_i + \mathbf{c}_j)/2$. The weight of each bridge, denoted as w_{ij}^{bridge}, is determined proportionally to the bidirectional transition flux between states. Specifically, it is calculated as:

$$w_{ij}^{\text{bridge}} = \eta \frac{\Phi_{ij}}{\Delta t} \tag{3}$$

where $\Phi_{ij} = \pi_i A_{ij} + \pi_j A_{ji}$ represents the transition flux in the stationary state, Δt is the time step of the discrete transition matrix A, and η is a scaling coefficient for adjusting the saddle point height. This formulation ensures that state pairs with larger transition fluxes are assigned larger weights w_{ij}^{bridge}, which lowers the potential barrier between the two stable points. This mechanism facilitates particle transport between pairs of states that exhibit frequent transitions.

Note that since Langevin dynamics is non-directional, the transition probability matrix A does not strictly match the transition probability matrix of the discrete Markov process obtained after clustering.

2.1.2 Experimental Settings for Data Generation

In the generation experiment, we assumed a system with three metastable states ($M = 3$) and arranged the center coordinates of each state to form the vertices of an equilateral triangle as follows:

$$\text{State 0} : (0, 1), \quad \text{State 1} : \left(\frac{\sqrt{3}}{2}, -\frac{1}{2}\right), \quad \text{State 2} : \left(-\frac{\sqrt{3}}{2}, -\frac{1}{2}\right) \tag{4}$$

To generate datasets with different transition dynamics, we used the following two types of transition probability matrices, A_1 and A_2:

$$A_1 = \begin{pmatrix} 0.8 & 0.2 & 0.0 \\ 0.2 & 0.7 & 0.1 \\ 0.0 & 0.1 & 0.9 \end{pmatrix}, \quad A_2 = \begin{pmatrix} 0.75 & 0.25 & 0.0 \\ 0.25 & 0.5 & 0.25 \\ 0.0 & 0.25 & 0.75 \end{pmatrix} \tag{5}$$

Simulation parameters were set as follows: variance $\sigma = 0.3$, temperature $k_B T = 0.15$, time step width $\Delta t = 0.1$, and scaling coefficient $\eta = 1.0$.

For each transition probability matrix, $N = 1000$ initial values $\mathbf{x}_0$ were sampled from a standard normal distribution $\mathcal{N}(0, I)$, and numerical integration was performed using the Euler-Maruyama method over the time interval $t \in [0, 120]$. To eliminate dependence on the initial state and obtain data in the stationary state, the first 200 steps were removed as a burn-in period.

The actual potential landscape and representative generated trajectories are shown in Figure 1.

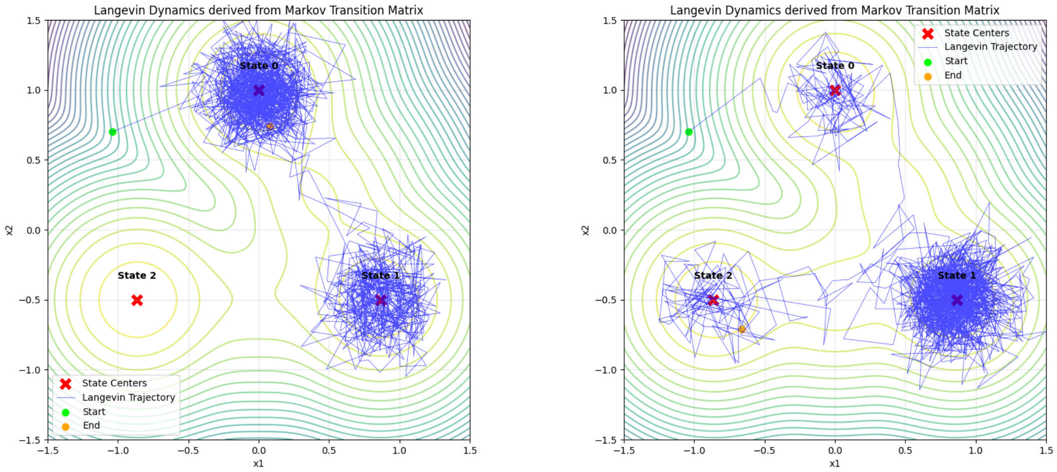

(a) A_1 (b) A_2

Figure 1: Potential landscape and generated representative trajectories.The data used for learning is a mixture of time series data generated from two Langevin dynamics.

The final observed data was obtained by downsampling the simulation results for A_1 and A_2 at a rate of 10, formatted as a 3D tensor data $\mathcal{D} \in \mathbb{R}^{2N \times T \times 2}$ with temporal correlation. Furthermore, to verify the robustness of the method against missing data, we applied a mask following a Bernoulli distribution $B(1, p)$ to the generated complete data, creating datasets with missing values under each condition of observation probability $p \in \{0.5, 0.6, 0.7, 0.8, 0.9, 1.0\}$. Note that the missing rate is $1 - p$.

In addition to the Missing Completely At Random (MCAR) scenario, we also conducted a validation in a scenario approximating Missing Not At Random (MNAR), where the missing rate varies depending on the state. The generated time-series data were spatially partitioned into three regions, C_0, C_1, C_2, using the k-means algorithm, and distinct observation probability vectors were assigned to each region.

The configured observation probabilities are as follows:

- Pattern 1: $[P_{C_0}, P_{C_1}, P_{C_2}] = [0.2, 0.6, 1.0]$
- Pattern 2: $[P_{C_0}, P_{C_1}, P_{C_2}] = [0.4, 0.6, 0.8]$
- Pattern 3: $[P_{C_0}, P_{C_1}, P_{C_2}] = [0.6, 0.6, 0.6]$

- Pattern 4: $[P_{C_0}, P_{C_1}, P_{C_2}] = [0.8, 0.6, 0.4]$
- Pattern 5: $[P_{C_0}, P_{C_1}, P_{C_2}] = [1.0, 0.6, 0.2]$

2.2 Application of the Proposed Method

Under the same design conditions as the proposed method, we trained the Deep State-Space Model, generated latent states, and performed clustering using k-means. Furthermore, we conducted 5 experiments with different seeds to check for statistical validity. Detailed settings are shown in Table 1.

Table 1: Hyperparameters and Experimental Settings.

Parameter Name	Description	Value
Dim	Dimension of latent space	8
Lr	Learning rate	1×10^{-2}
Batch_Size	Batch size	100
Epoch	Number of epochs	50
K	Number of clusters for k-means	5

2.3 Evaluation Metrics

To quantitatively evaluate the validity of the clustering structure possessed by the latent representations extracted by the proposed method, we adopted the Adjusted Rand Index (ARI) and Normalized Mutual Information (NMI), which are widely used as supervised clustering evaluation metrics. These metrics measure the degree of agreement between the clustering results of the reference ground truth (or baseline dataset) and the clustering results to be evaluated.

2.3.1 Adjusted Rand Index (ARI)

The Rand Index (RI) evaluates the similarity between two data partitions (clustering results) U and V based on the co-occurrence relationship of data pairs. For all pairs formed from n data samples (${}_nC_2$), it counts the matches for the following two conditions:

- Pairs belonging to the same cluster in U also belong to the same cluster in V.
- Pairs belonging to different clusters in U also belong to different clusters in V.

However, RI tends to take positive values even for random clustering and increases as the number of clusters increases, making fair comparison under different conditions difficult. The Adjusted Rand Index (ARI) normalizes RI by subtracting the expected index due to chance. ARI is defined as follows:

$$\mathrm{ARI} = \frac{\mathrm{RI} - E[\mathrm{RI}]}{\max(\mathrm{RI}) - E[\mathrm{RI}]} \tag{6}$$

Here, $E[\mathrm{RI}]$ is the expected value of RI under random partitioning, and $\max(\mathrm{RI})$ is the maximum possible value of RI. An ARI of 1.0 indicates a perfect match, while values near 0.0 (or negative values) imply equivalence to random labeling. This property allows for fair comparison even when cluster numbers or sample sizes differ.

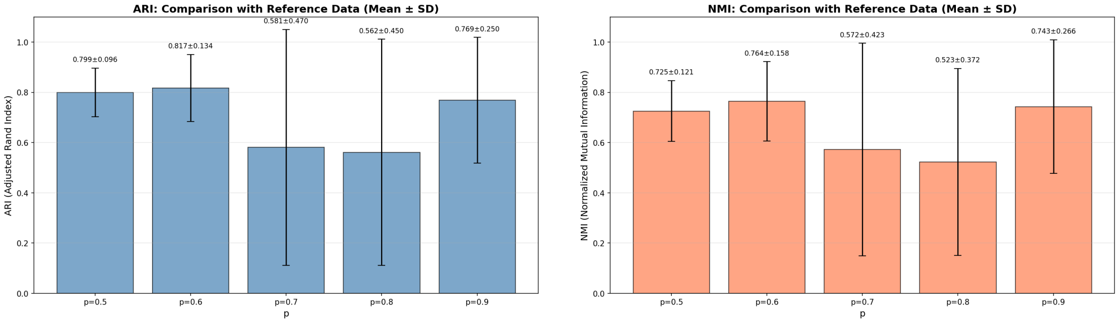

(a) ARI (b) NMI

Figure 2: Clustering performance metrics across different observation probabilities on the MCAR scenario. Left: Adjusted Rand Index (ARI). Right: Normalized Mutual Information (NMI). Error bars indicate standard deviation over 5 random seeds.

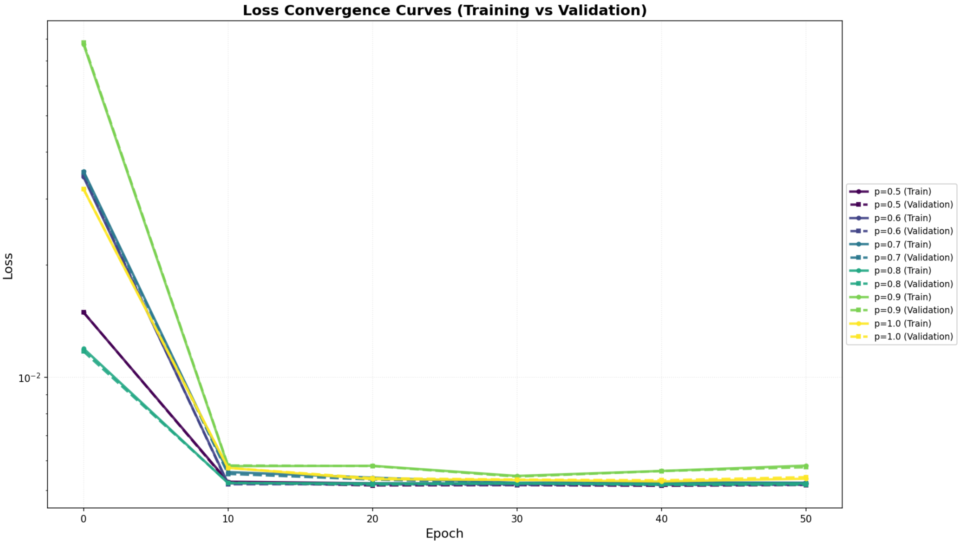

Figure 3: Learning curves (Training and Validation Loss) across different observation probabilities (p).

2.3.2 Normalized Mutual Information (NMI)

Normalized Mutual Information (NMI) is an evaluation metric based on information theory that normalizes the Mutual Information (MI) between two clustering results U and V by their respective entropies. First, the entropies $H(U), H(V)$ of clustering results U, V and the mutual information $I(U, V)$ are defined as follows:

$$H(U) = -\sum_i P(u_i) \log P(u_i) \tag{7}$$

$$I(U, V) = \sum_i \sum_j P(u_i, v_j) \log \frac{P(u_i, v_j)}{P(u_i)P(v_j)} \tag{8}$$

Here, $P(u_i)$ represents the probability that a sample belongs to cluster u_i, and $P(u_i, v_j)$ represents the joint probability of belonging to cluster u_i and v_j simultaneously. Mutual information $I(U, V)$ represents how much uncertainty about U is reduced (information gained) by knowing V. However, since it tends to increase with the number of clusters, it is not suitable for comparison as is.

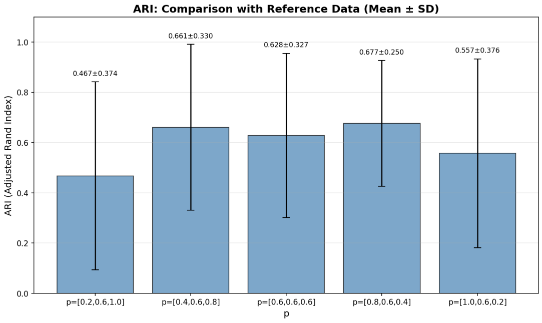

(a) ARI

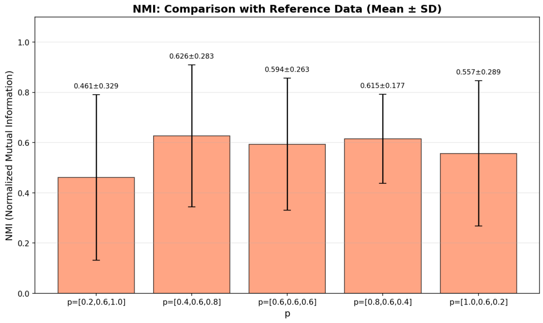

(b) NMI

Figure 4: Clustering performance metrics across different observation probabilities on the MNAR scenario. Left: Adjusted Rand Index (ARI). Right: Normalized Mutual Information (NMI). Error bars indicate standard deviation over 5 random seeds.

NMI is a metric normalized to the range of 0 to 1 by using the geometric or arithmetic mean of the mutual information. In this study, we use the following definition:

$$\mathrm{NMI}(U, V) = \frac{2 \cdot I(U, V)}{H(U) + H(V)} \tag{9}$$

An NMI of 1.0 indicates that the two clustering results share information completely (perfect agreement), while a value closer to 0.0 indicates that they are independent (unrelated). Compared to ARI, NMI is often considered to be more sensitive to the distribution shape of clusters.

2.3.3 Evaluation Protocol

In our experimental protocol, we established a quantitative framework to assess structural consistency of the learned latent states. We designated the latent states derived from the fully observed dataset (no masking, $p = 1.0$) as the reference representation. Baseline cluster labels, denoted as L_{ref}, were generated by applying K-means clustering with $K = 3$ to these reference states. To evaluate robustness against data sparsity, we performed identical clustering on latent states inferred from datasets with varying observation probabilities (MCAR and MNAR scenario), yielding target labels L_{target}. The degree of structural preservation was then quantified by computing the ARI and NMI metrics between L_{ref} and the respective L_{target} for each probability. All evaluations were aggregated over five independent training runs initialized with different random seeds to account for stochastic variability in the Deep State-Space Model.

3 Results

Under the above conditions, we investigated the validity of clusters at each observation probability compared to clusters without missing data based on ARI and NMI. Figure 2 shows the differences in ARI and NMI at each observation rate, with error bars representing the deviation from 5 experiments.

The proposed method, based on variational inference, exhibited large variance due to posterior collapse. However, the impact of missing time-series data was small, and the dependency between classes was sufficiently clear even under the condition of $p = 0.5$, which is the data setting of this proposal.

In addition, to confirm the stability and robustness of model training, we analyzed the learning curves for each observation probability $p \in \{0.5, \ldots, 1.0\}$ (Figure 3). As shown in Figure 3, the Training loss and Validation loss showed similar convergence behavior under all

conditions, and no significant divergence was observed between them. This suggests that the model is able to learn stably without overfitting, even under conditions with a high missing rate ($p = 0.5$).

Finally, similar experiments were conducted for the MNAR scenario, yielding the results shown in Fig. 4. Consistent with the MCAR scenario, the proposed method based on variational inference also exhibited large variance depending on the initialization of the neural network.

4 Conclusion

From the above results, it was confirmed that even in an environment with a high missing rate ($p = 0.5$), the proposed method (1) can appropriately recover the structure of latent states (clustering), (2) has a stable learning process, and (3) has a low risk of overfitting. Consequently, concerns regarding bias and instability of initial state estimation caused by high missing rates in real data (EHR) are considered to be limited in this method.
