# Supplementary material for: A deep state-space analysis framework for cancer patient latent state estimation and classification from EHR time-series data
Source: PLoS One. 2026 Jan 30;21(1):e0341003. doi: 10.1371/journal.pone.0341003 (PMC12858016; doi:10.1371/journal.pone.0341003)
Supplement: S4 Appendix — This sensitivity analysis confirms that the patient clustering results are robust to changes in UMAP hyperparameters. (PDF) [file pone.0341003.s006.pdf]

S6 Appendix. Sensitivity Analysis of UMAP Hyperparameters

Yuji Okamoto, Aya Nakamura, Ryosuke Kojima, Eiichiro Uchino,
Yohei Mineharu, Yohei Harada, Mayumi Kamada, Minoru Sakuragi,
Manabu Muto, Motoko Yanagita, Yasushi Okuno

Article Title: A Deep state-space Analysis Framework for Cancer Patient Latent State Estimation and Classification from EHR Time-Series Data

1. Objective

In the main manuscript, Uniform Manifold Approximation and Projection (UMAP) was employed to visualize the high-dimensional latent states estimated by the Deep State-Space Model (DSSM) in a two-dimensional space. To verify the robustness of the observed patient clusters (Dangerous, Intermediate, and Stable states), we conducted a sensitivity analysis by varying the `n_neighbors` parameter, which controls the balance between local and global structure preservation.

2. Methods

Dimensionality reduction was performed using UMAP with the **`n_neighbors`** parameter varied to the following values:

1. $n = 5$
2. $n = 10$
3. $n = 15$ (Selected parameter used in the main manuscript)
4. $n = 20$

All other UMAP parameters were kept at their default settings.

3. Results

Figure A displays the 2D scatter plots of patient latent states at the final time points for the tested `n_neighbors` values.

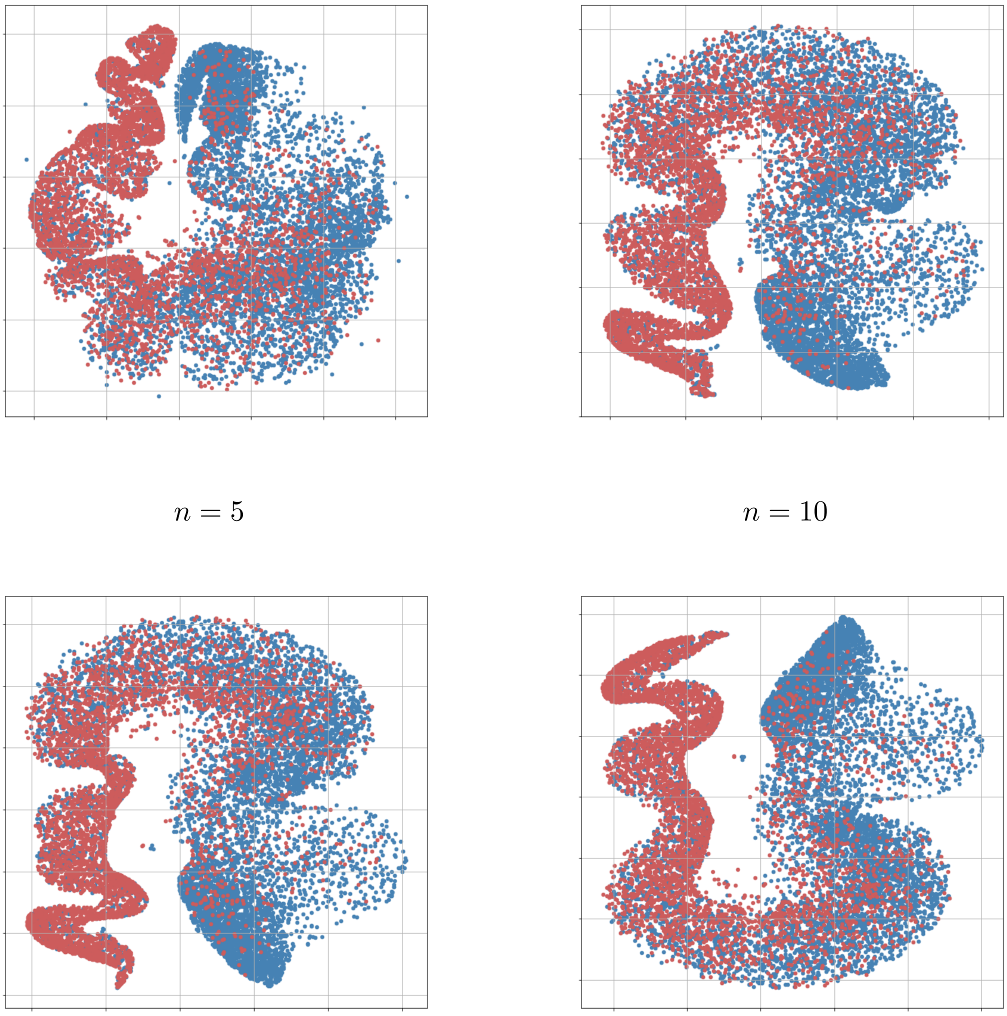

$n = 5$ $n = 10$

$n = 15$ (This paper used.) $n = 20$

Figure 1: **UMAP visualization with varying `n_neighbors`.** The scatter plots show the latent states of patient endpoints for Deceased (Red) and Surviving (Blue) patients. Panels correspond to `n_neighbors` values of (a) 5, (b) 10, (c) 15, and (d) 20. The separation between the two groups is robust across these parameter settings.

- **Consistency:** Across all settings ($n = 5, 10, 15, 20$), the spatial separation between deceased patients (red) and surviving patients (blue) remained consistent.
- **Structure:** The gradient from the "Stable" state to the "Dangerous" state via the "Intermediate" state was preserved in all visualizations, indicating that the identified clinical states are intrinsic to the data rather than artifacts of a specific parameter choice.

4. Conclusion

The sensitivity analysis confirms that the structural distinction between surviving and deceased patients is robust to changes in the parameters. The choice of $n = 15$ in the main text is therefore considered appropriate and representative.
