# Supplementary material for: A deep state-space analysis framework for cancer patient latent state estimation and classification from EHR time-series data
Source: PLoS One. 2026 Jan 30;21(1):e0341003. doi: 10.1371/journal.pone.0341003 (PMC12858016; doi:10.1371/journal.pone.0341003)
Supplement: Attachment — Submitted filename: Respons_to_reviewers_PlosONE_deepssm.pdf [file pone.0341003.s010.pdf]

Response to Reviewers

Manuscript ID: PONE-D-25-02277R2
Title: A Deep state-space Analysis Framework for Cancer Patient Latent State Estimation and Classification from EHR Time-Series Data

Dear Editor and Reviewers,

We would like to express our sincere gratitude for the further constructive comments provided by Reviewer #2. These suggestions have been incredibly helpful in strengthening the validation of our model's reliability and robustness. In this revision, we have conducted additional analyses using simulated data ("toy data") and sensitivity analyses for UMAP visualization to address the concerns regarding model stability, missing values, and parameter dependence. We have added these results as new Appendices.

Below is our point-by-point response to the specific comments.

Response to Reviewer #2

Comment 1:
The authors appear to attempt to include both clinical and technical aspects in the manuscript, but the emphasis is clearly more on the technical side. Therefore, the clinical information regarding drug usage, control conditions, and medical tests should be briefly but clearly described. It would be important to explain why only the drugs or factors/medical tests mentioned in the text were included. As it currently stands, readers must accept these choices at face value without sufficient supporting justification. This issue is particularly relevant because many potential factors could be considered in such analyses. While omitting certain medical tests may improve model performance, such omissions may not be clinically appropriate or justifiable.

Response:
Thank you for pointing out the need for clearer clinical context. We agree that the selection criteria for drugs and medical tests were not sufficiently explained. Specifically, regarding the 50 clinical laboratory test items, as noted in the figure captions, we selected items that show significant fluctuation due to drug administration (items with a high frequency of abnormal values). Regarding the anticancer drugs, we selected the top 8 drugs with the highest usage (prescription volume) at our institution. We have added a clear description of these selection criteria to the revised **Methods** section to clarify that these choices were based on data characteristics and clinical frequency rather than arbitrary selection. Additionally, we have included the number of patients administered each drug in the Appendix.

Comment 2:
The comparison with other approaches should also specify how those methods were framed and implemented, not merely their final results.

Response:
We appreciate this suggestion. We have expanded the description of the comparison methods (PCA, VAE, Linear State-Space Model) in the **Methods** section and **S2 Appendix**. We explicitly described the network architecture, loss functions, and training procedures for these baseline models to ensure fair and transparent comparison. This allows readers to understand that the performance differences stem from the model's ability to capture non-linear temporal dynamics rather than implementation disparities.

Comment 3:
An existing time-series model was used, trained, and tested with current data. However, this part is not clearly described in the manuscript. If the model was not retrained, it should be explicitly stated that

testing was performed directly on existing data. In that case, an analysis of the model's applicability and validity under these conditions is needed.

Response:
We apologize for the ambiguity in our description. The main objective of this study is **unsupervised latent state estimation and clustering**, rather than building a prediction model using supervised learning. Therefore, we do not apply a method of splitting data into training and test sets to evaluate prediction accuracy; instead, the final output results are applied to and visualized for all data, including the training data. However, during the Neural Network training process, we utilized a Validation set for hyperparameter tuning. In this study, we estimated the latent states of the entire patient dataset using the model adjusted in this manner and analyzed its structure. We have explicitly stated in the **Methods** section that we used validation for adjustment and performed the analysis on the full dataset to clarify this approach.

Comment 4:
A high proportion of missing values (>50%) may cause instability in latent state estimation, particularly during the early stages of the time series. It should be analyzed whether the imputation procedure introduced bias, especially for laboratory measurements with large temporal fluctuations. The initial latent state estimation heavily depends on the imputed values; therefore, substantial imputation bias can affect model convergence and degrade the quality of the estimated latent states. The stability and convergence of the model training process should be demonstrated, particularly given the high proportion of missing values. It would also be helpful to include additional quantitative metrics to assess model stability and robustness.

Response:
These are extremely important points. To address the concern that high missing rates might introduce bias or instability, we conducted a robustness validation using ”toy data” (simulated data) where the ground truth is known. We generated synthetic time-series data with latent state transitions and introduced missing values at rates comparable to our real EHR dataset. We then applied our Deep State-Space Model to this dataset. The results demonstrated that our model successfully recovered the true latent structure even with high rates of missingness, confirming that our handling of missing values (masking loss function combined with initial imputation) does not significantly degrade the estimation quality. Furthermore, to verify the stability of the training, we confirmed the learning curves showing the convergence of the loss function. In addition, we demonstrated that the clustering results are consistent across different random seeds, quantitatively supporting the model's stability despite the sparse nature of EHR data. We have included these combined analysis results in a new **S5 Appendix. (Robustness of DeepSSM to Missing Data Rates.)**. Accordingly, we have updated the Methods section (under 'Deep state-space model') in the revised manuscript to explicitly cite S5 Appendix, ensuring readers are aware of this robustness validation.

Comment 5:
The robustness validation of the UMAP results would also be helpful.

Response:
Thank you for this suggestion. To verify that the identified patient states (Dangerous, Intermediate, Stable) are not artifacts of specific UMAP hyperparameters, we performed a sensitivity analysis. We present the changes in the latent space when varying the key UMAP parameter, 'n_neighbors' (ranging from 5 to 20), in **S6 Appendix (UMAP Robustness Analysis)**. The results showed that the global structure remained consistent against parameter changes. This confirms that the visualization reflects the intrinsic structure of the high-dimensional latent space learned by the model, rather than being a result of parameter tuning. We have also revised the Methods section (under 'Visualization of latent states') to include a reference to this sensitivity analysis (S6 Appendix), clarifying the stability of our clustering results.

Comment 6:
The paper analyzes all types of cancer; how are the results for specific single cancer subtypes?

Response:
We acknowledge the importance of analyzing specific cancer subtypes. While our current study aimed to establish a generalizable framework applicable across diverse cancer types, we understand the clinical interest in subtype-specific trends. However, subdividing the dataset into specific single cancer types results in smaller sample sizes for many groups, making deep learning-based estimation unstable with the current configuration. We have added a statement to the **Limitations** in the **Discussion** section that analysis by specific subtypes is difficult with the current dataset due to insufficient sample sizes for specific single cancer types, and we have noted this as a task for future research.

We believe these additional validations and revisions have addressed the concerns regarding the robustness and clinical context of our study.

Sincerely,

Yasushi Okuno, Ph.D.
Department of Biomedical Data Intelligence
Graduate School of Medicine, Kyoto University
