# Supplementary material for: A deep state-space analysis framework for cancer patient latent state estimation and classification from EHR time-series data
Source: PLoS One. 2026 Jan 30;21(1):e0341003. doi: 10.1371/journal.pone.0341003 (PMC12858016; doi:10.1371/journal.pone.0341003)
Supplement: Attachment — Submitted filename: Respons_to_reviewers_PlosONE_deepssm_v5.pdf [file pone.0341003.s011.pdf]

# Response to Reviewers

**Manuscript ID:** PONE-D-25-02277R2
**Title:** A Deep state-space Analysis Framework for Cancer Patient Latent State Estimation and Classification from EHR Time-Series Data

We would like to express our sincere gratitude to the Academic Editor and Reviewer #2 for their continued evaluation of our manuscript. We appreciate the positive feedback and the insightful comments regarding the consistency of our descriptions and the interpretation of our robustness analysis.

We have revised the manuscript to address the specific points raised by Reviewer #2. Below is our point-by-point response to the comments.

# Response to Reviewer #2

**Comment 1:**
The reported transitions between clusters for deceased and surviving patients (lines 359–361) appear inconsistent with the figure, and the authors should clarify this discrepancy.

**Response:**
Thank you for pointing out this discrepancy. We carefully re-examined our data and found that the transition probabilities reported in the Supplementary Table (S3 Table) were correct, but Figure 2d was incorrect.

Accordingly, we have revised **Figure 2d** to accurately reflect the correct transition probabilities. The figure is now consistent with the descriptions in the text and the data in the Supplementary Materials.

**Comment 2:**
The robustness analysis using SDE-based synthetic data and ARI/NMI metrics is useful, but it should be noted that it only covers MCAR scenarios and may not fully reflect real-world EHR missingness patterns.

**Response:**

We agree that real-world EHR data often involve complex missingness mechanisms beyond Missing Completely At Random (MCAR). To address this concern and further validate our model's robustness, we conducted additional experiments using **Missing Not At Random (MNAR) scenarios**, where the probability of missing data depends on the latent state. We specifically adopted state-dependent MNAR scenarios because it is well-known in medical data that the probability of missing values often varies depending on the patient's severity or condition (latent state)[1].

Our results confirmed that the model maintains a similar level of accuracy in MNAR scenarios as it does in MCAR scenarios. We have added these new findings to S5 Appendix.

[1] Weiskopf NG, Rusanov A, Weng C. Sick patients have more data: the non-random completeness of electronic health records. AMIA Annu Symp Proc. 2013 Nov 16;2013:1472-7. PMID: 24551421; PMCID: PMC3900159.

We believe these revisions have resolved the remaining issues and improved the accuracy and transparency of our manuscript. Thank you again for your valuable time and guidance.

Sincerely,

Yasushi Okuno, Ph.D.
Department of Biomedical Data Intelligence
Graduate School of Medicine, Kyoto University
