# Supplementary material for: A deep state-space analysis framework for cancer patient latent state estimation and classification from EHR time-series data
Source: PLoS One. 2026 Jan 30;21(1):e0341003. doi: 10.1371/journal.pone.0341003 (PMC12858016; doi:10.1371/journal.pone.0341003)
Supplement: Attachment — Submitted filename: Response_to_reviewers.docx [file pone.0341003.s008.docx]

# Response to the Editor and Reviewers

**Manuscript title (revised)**
**“A Deep State‑Space Analysis Framework for Estimating Latent States in Cancer Patients from EHR Time‑Series Data”**

**Authors**

Yuji Okamoto,^1*^ Aya Nakamura,^1*^ Ryosuke Kojima,^1*^ Eiichiro Uchino,^1,2^ Yohei Mineharu,^1,3^ Yohei Harada,^1^ Mayumi Kamada,^1^ Minoru Sakuragi,^1,2^ Manabu Muto,^4^ Motoko Yanagita,^2,5^ Yasushi Okuno^1^

^1^ Department of Biomedical Data Intelligence, Graduate School of Medicine, Kyoto University

^2^ Department of Nephrology, Graduate School of Medicine, Kyoto University

^3^ Department of Artificial Intelligence in Healthcare and Medicine, Graduate School of Medicine, Kyoto University

^4^ Department of Therapeutic Oncology, Graduate School of Medicine, Kyoto University

^5^ Institute for the Advanced Study of Human Biology (ASHBi), Kyoto University

^*^Equal Contribution

We appreciate the time and effort the Editor and the six anonymous reviewers have devoted to evaluating our work. Below we address every point raised. Reviewer comments are reproduced **in bold italics**, followed by our point‑by‑point **responses in plain text**. All page, line, figure and table numbers cited below refer to the newly revised manuscript with tracked changes.

## Response to the Editor

Thank you for noting that funding information should appear only in the cover letter. As requested, we have removed this text from the main manuscript and placed it in the cover letter.

## Global Revisions Undertaken (all reviewers)

1. **Manuscript structure** – The Methods section now precedes Results and Discussion (pp. 5–14), the Abstract has been converted to PLOS ONE’s structured format, and terminology has been standardised throughout.
2. **Title** – Revised as shown above to reflect patient population and omit the word “new.”
3. **Methodological transparency** –
   - Missing‑data handling, abnormal‑value flags, UMAP settings, hyper‑parameter search and imputation methods are now detailed in §2.3–2.5 (pp. 7–11) and Table S3.
   - A concise statement of our three methodological contributions has been added to the end of the Introduction (p. 4).
4. **Evaluation policy** – Because the study is fully unsupervised, we added a paragraph (p. 15) explaining why standard quantitative metrics are inappropriate and why qualitative clinical validation is emphasised.
5. **Figures and tables** – Axis labels, legends and explanatory captions have been added or expanded for every figure; Figure 1’s workflow has been redrawn to show the correct order (UMAP after clustering).
6. **Citation style** – Converted to Vancouver style in compliance with PLOS ONE guidelines.
7. **Language** – The manuscript has been shortened by ~15 %, redundant sentences removed and wording tightened for clarity.

## Detailed Response to Reviewer 1

**1. Innovative methodology & missing‑data handling need clarification.**
We now describe (pp. 7–9) how missing values are (i) initially imputed with a zero‑order spline and (ii) further refined by sampling from the decoder during training. Extended Data 4 lists the grid‑search ranges used for hyper‑parameters and explains why qualitative validity was adopted in lieu of a single tuning criterion.

**2. Selection bias from excluding patients with < 50 time steps; temporal shifts (2006–2018).**
A justification based on the empirical distribution in new Extended Figure 2 and an explicit note on pp. 6–7 now clarify this threshold. We also discuss potential temporal drift in EHR coding and practice (p. 18, limitations).

**3. Causal interpretation & cancer‑type heterogeneity.**
We added cautionary language (p. 16) emphasising that risk factors are correlative, not causal, and explained limitations arising from uncertain tumour‑type metadata. Drug‑specific analyses are retained in Table 2 and Supplementary Table S5.

## Detailed Response to Reviewer 2

**1. Terminology consistency & scientific language.**
The entire manuscript was line‑edited; examples of changed terms include “proposed framework” → “presented framework” (Abstract, p. 2) and “in‑model learning” → “decoder‑based imputation” (§2.3).

**2. Manuscript structure (Methods before Results).**
Implemented as requested (see Global Revision 1).

**3. Undefined categorisation criteria (abnormal high/low).**
Section 2.2 (pp. 6–7) now explains that each laboratory record in the raw EHR carries an institutional flag relative to its sex‑, age‑ and period‑specific reference range, mirroring CTCAE guidelines.

**4. Differentiate existing methods from our contributions.**
A new paragraph at the end of the Introduction (p. 4) itemises our three contributions. All model components are clearly cited to Fraccaro et al. (2017) [ref 13] where unchanged.

**5. Clarity of methodological description, UMAP parameters, cluster labels.**
Section 2.4 (p. 10) lists the UMAP parameters (n _neighbours = 15, min_dist = 0.1; all else default) and notes that varying them did not materially alter the visualisation. The mapping between cluster labels I–III and latent states z_t has been added in §2.5 (p. 11).

## Detailed Response to Reviewer 3

**1. Novelty of anaemia finding.**
We clarified in the Introduction (p. 4) that the study aims to confirm that established risk factors such as anaemia can be retrieved de novo from longitudinal EHR data, thereby validating the framework rather than proposing new biology.

**2. Communication to clinicians & trustworthiness.**
The Discussion (p. 17) now highlights that the model relies on minimal hand‑engineered preprocessing, reducing bias, and that the latent‑state visualisations provide an intuitive bridge for non‑experts.

**3. Clinical deployment & reproducibility.**
A paragraph on future integration into clinical decision‑support tools has been added (p. 19). The GitHub repository has been updated; README now explicitly links to an expanded synthetic dataset.

## Detailed Response to Reviewer 4

**1. Causal inference claim (lines 248–254).**
We removed the misleading sentence and now explicitly state that the framework is retrospective and observational; causal hypotheses require prospective validation (p. 15).

**2. Missing‑rate calculation & robustness to high missingness.**
Section 2.3 (p. 8) defines the per‑variable missing rate (mean 41 %, range 3–89 %) and explains how the mask and decoder jointly handle extensive missingness.

**3. Discrepancy in Table 2 counts.**
Table 2 has been corrected; patients with multiple ICD‑10 codes are now counted in each applicable row, hence the sum exceeds the total cohort.

**4. Order of UMAP and K‑means in Figure 1.**
Figure 1 has been redrawn; caption revised to state that K‑means clustering precedes 2‑D UMAP projection.

**5. Interpretation of transition probabilities (Figure 2d).**
We now explain (pp. 13–14) that absolute differences between opposing arrows are small and not themselves indicative; rather, the higher frequency of transitions among deceased patients concentrates their trajectories in the ‘dangerous’ state.

**6. Death definition, latent‑state dimensionality, MinMax & spline methods, incomplete sentence (l. 439).**
All points have been corrected or clarified in §2.1, §2.3 and §4.1.

## Detailed Response to Reviewer 5

**1. Title, structured abstract & wording (lines 23, 74).**
Implemented (see Global Revisions 1–3). “Identified framework” now replaces “proposed” at the first mention (Abstract). The specific framework is named at each reference.

**2. Conciseness & citation style.**
The manuscript has been shortened and all citations converted to Vancouver style as requested.

**3. Methods before Results; distinguish prior vs. current model; add figure.**
Structural change implemented. A schematic in new Figure S1 contrasts the original deep state‑space model with our EHR‑adapted workflow.

## Detailed Response to Reviewer 6

**1. Lack of quantitative validation.**
We expanded §4.2 (p. 16) to explain why no widely accepted quantitative benchmark exists for unsupervised, non‑linear latent‑state clustering of EHR time‑series and why qualitative clinical validation is the current standard.

**2. State‑transition differences by cancer type, sex, age.**
We focus on eight index drugs as stated in §1.2 and §3.4; subgroup analyses by tumour type or demographic strata are outside the scope of this manuscript but flagged as future work (p. 18).

**3. Comparison with other embedding methods.**
We added a short experiment (Supplementary Figure S6) comparing PCA, t‑SNE and our approach; PCA and t‑SNE produced mixed clusters with poor clinical interpretability, supporting our choice of the state‑space model.

**4–6. Figure clarity, bubble‑plot legend, literature support.**
All figure captions expanded; bubble‑plot size is now explained as the cluster‑specific proportion of each laboratory flag. Additional supporting citations have been added in §4.3.

## Concluding Remarks

We believe that the manuscript is now substantially clearer, more concise and scientifically rigorous, and we hope that the revisions satisfactorily address every concern. We thank the Editor and reviewers once again for their invaluable guidance.
