# Supplementary material for: A deep state-space analysis framework for cancer patient latent state estimation and classification from EHR time-series data
Source: PLoS One. 2026 Jan 30;21(1):e0341003. doi: 10.1371/journal.pone.0341003 (PMC12858016; doi:10.1371/journal.pone.0341003)
Supplement: Attachment — Submitted filename: Response_to_reviewers_v3.docx [file pone.0341003.s009.docx]

# PLOS ONE Response to Reviewers

Manuscript Title: A Deep state-space Analysis Framework for Cancer Patient Latent State Estimation and Classification from EHR Time-Series Data

Authors: Yuji Okamoto,^1*^ Aya Nakamura,^1*^ Ryosuke Kojima,^1*^ Eiichiro Uchino,^1,2^ Yohei Mineharu,^1,3^ Yohei Harada,^1^ Mayumi Kamada,^1^ Minoru Sakuragi,^1,2^ Manabu Muto,^4^ Motoko Yanagita,^2,5^ Yasushi Okuno^1^

^1^ Department of Biomedical Data Intelligence, Graduate School of Medicine, Kyoto University

^2^ Department of Nephrology, Graduate School of Medicine, Kyoto University

^3^ Department of Artificial Intelligence in Healthcare and Medicine, Graduate School of Medicine, Kyoto University

^4^ Department of Therapeutic Oncology, Graduate School of Medicine, Kyoto University

^5^ Institute for the Advanced Study of Human Biology (ASHBi), Kyoto University

^*^Equal Contribution

Manuscript ID: PONE-D-25-02277R1

To the Editor-in-Chief, Dr. Emily Chenette, the Handling Editor, and the Reviewers of PLOS ONE

We would like to express our sincere gratitude for the constructive and detailed comments on our manuscript, "A Deep state-space Analysis Framework for Cancer Patient Latent State Estimation and Classification from EHR Time-Series Data." We sincerely appreciate your taking the time to review our work. We have carefully considered all the points you raised and have made substantial revisions throughout the manuscript to enhance its quality.

Below, we provide a point-by-point response to each of the reviewers' comments and detail the corresponding revisions made to the manuscript.

### Response to Reviewer #2

**Comment 1: Cluster I contains ~39% of surviving patients, and Cluster II contains ~36% of deceased patients. The manuscript defines Cluster II as an intermediate state, but it is unclear why Cluster I is not also considered intermediate?**

Response:

Thank you for this insightful question regarding our cluster definitions. We apologize that our explanation was insufficient.

Our cluster definition places significant weight on the distribution of patients at their **final time point** of observation, rather than their distribution throughout the entire observation period. When we analyze the data focusing specifically on these final time points, a clear trend emerges: a majority of deceased patients end their observation period in Cluster I, while a majority of surviving patients end in Cluster III. In contrast, Cluster II contains a mixture of final time points for both deceased and surviving patients and also serves as a hub that many patients transition through. For these reasons, we defined it as the "intermediate state."

To clarify this logic, we have revised the **Results** section, specifically in the description of Figure 2, to explicitly state that Cluster I is defined as the "dangerous state" and Cluster III as the "stable state" based on the distribution at the final time point.

**Comment 2: The reliability of the proposed model needs to be addressed.**

Response:

Thank you for pointing out the need to address the model's reliability. We have strengthened the manuscript to better support the reliability of our model. Specifically, we have elaborated on three aspects in the Discussion section: (1) The superiority of our model in clearly separating clinical outcomes (survival vs. death) compared to models that do not consider temporal continuity, such as PCA and VAE (Figure 4). (2) The fact that the risk factors extracted by our model in a data-driven manner, such as anemia, are strongly consistent with existing clinical knowledge, suggesting that the model accurately captures the clinical reality. (3) The implementation of grid search for hyperparameter tuning to optimize model performance. We believe these multifaceted validations collectively ensure the reliability of our framework.

**Comment 3: The parameters presented in tables and figures need clearer definitions and explanations, either in clinical or theoretical terms. Readers unfamiliar with the domain may not fully understand their significance without further context.**

Response:

Thank you for this suggestion. To ensure our research is accessible to readers from various fields, we have carefully reviewed all figures and tables and have substantially expanded their captions to provide clearer definitions.

**Comment 4: The manuscript would benefit from clearer writing, particularly in explaining the clinical implications and the interpretation of both data and model output.**

Response:

Thank you for this feedback. Reflecting on your comment, we have revised the entire manuscript, especially the Discussion section, to clarify the clinical significance and interpretation of our results. Specifically, we have shifted the focus from a purely technical explanation of the model's superiority (e.g., comparison with VAE) to emphasizing why and how this technical advantage translates into clinical value. For instance, instead of just stating that "the model's nonlinearity allowed for the discrimination of deceased and surviving patients," we have rephrased this to highlight the clinical benefit: "the model can estimate highly interpretable states that allow clinicians to intuitively grasp a patient's prognosis." We believe this revision makes the significance of our research clearer.

### Response to Reviewer #3

**Comment 1: I still do not see any sample data in the GitHub repository. I do see mention of a sample.csv file, but there isn’t simulated or sample data to utilize with the code. Since this manuscript is largely based on a novel computational method. The methods need to be reproducible, and I cannot recommend for publication without this resolved.**

Response:

Thank you for this crucial point regarding reproducibility. We sincerely apologize that this was not adequately addressed. In response to your comment, we have comprehensively updated our GitHub repository. We have uploaded all the code used for the analysis, visualization, and post-processing in this study. However, as our research is based on real patient EHR data, we cannot make the original data public due to privacy concerns. Therefore, the provided code cannot be run as is. While generating statistically identical reproducible data is challenging due to the complex nature of EHR data, we have included a sample dataset (sample.csv) that mimics the data structure to allow for verification of the code's algorithmic behavior. We have also noted these points and the limitations on reproducibility in the Code Availability section of the manuscript.

**Comment 2: I am unsure of the contributions and impact of this work to existing deep-learning methods. I understand the utility of estimating latent states from time series data, however, findings stemming from these methods lack explainability and without comparison with another dataset, it is difficult to know if there are cohort/dataset level factors driving the identification of the latent states.**

Response:

Thank you for your comment regarding the contribution and impact of our work. To clarify this point, we first want to state that the Deep State Space model used in this study is an established and validated method in the field of deep learning; therefore, we do not claim a contribution in terms of the novelty of the model itself. Our contribution lies in presenting an analytical framework that applies this powerful model to complex real-world data—EHR time-series—and translates its output into clinically interpretable insights. Acknowledging this, in the Discussion section, we recognize the limitation that our study is based on data from a single institution, while also discussing the generalizability of the proposed framework. We argue that it can be applied to other cancer types, chronic diseases, or datasets from different medical institutions.

**Comment 3: I recommend that the authors consult with clinicians to highlight ways in which this method can improve the existing clinical paradigm of cancer treatment. For example, could the Markov transition probabilities be used in a clinical algorithm? How sure could they be that the latent state identified is probable?**

Response:

Thank you for this very constructive suggestion regarding clinical application. We have had further discussions with our clinician co-authors about the future applicability of this research. As a result, we concluded that prospective clinical application (such as real-time monitoring) is challenging at this stage, given the retrospective nature of our data analysis.

However, we reached a consensus on the significant potential of this framework for estimating factors for patient stratification in clinical trials and real-world practice. For instance, we envision that the "dynamic transition patterns" of latent states estimated by our method could be used as a novel stratification factor, in addition to conventional static biomarkers. This could potentially lead to a more precise identification of patient subgroups who are more likely to respond to treatment.

Based on this discussion, we have revised the Discussion section to focus on "estimation of stratification factors." We believe this revision shows that our research is not limited to presenting a basic computational method but also has specific and feasible potential to contribute to the design of future clinical research.

This concludes our responses to the reviewers' comments and an overview of the revisions made. We are grateful for the valuable feedback, which has significantly improved the quality of our manuscript.
